# Down-regulated HHLA2 enhances neoadjuvant immunotherapy efficacy in patients with non-small cell lung cancer (NSCLC) with chronic obstructive pulmonary disease (COPD)

**DOI:** 10.1186/s12885-024-12137-5

**Published:** 2024-03-29

**Authors:** Ao Zeng, Yanze Yin, Zhilong Xu, Abudumijiti Abuduwayiti, Fujun Yang, Mohammed Saud Shaik, Chao Wang, Keyi Chen, Chao Wang, Xinyun Fang, Jie Dai

**Affiliations:** 1 Department of Thoracic Surgery, Shanghai Pulmonary Hospital, School of Medicine, Tongji University, 200433 Shanghai, China; 2 School of Medicine, Tongji University, 200092 Shanghai, China

**Keywords:** Non-small cell lung cancer, Chronic obstructive pulmonary disease, Neoadjuvant immunotherapy, HERV–H LTR-associating protein 2, Tissue-resident memory T cells

## Abstract

**Background:**

Emerging data suggested a favorable outcome in advanced non-small cell lung cancer (NSCLC) with chronic obstructive pulmonary disease (COPD) patients treated by immunotherapy. The objective of this study was to investigate the effectiveness of neoadjuvant immunotherapy among NSCLC with COPD versus NSCLC without COPD and explore the potential mechanistic links.

**Patients and methods:**

Patients with NSCLC receiving neoadjuvant immunotherapy and surgery at Shanghai Pulmonary Hospital between November 2020 and January 2023 were reviewed. The assessment of neoadjuvant immunotherapy’s effectiveness was conducted based on the major pathologic response (MPR). The gene expression profile was investigated by RNA sequencing data. Immune cell proportions were examined using flow cytometry. The association between gene expression, immune cells, and pathologic response was validated by immunohistochemistry and single-cell data.

**Results:**

A total of 230 NSCLC patients who received neoadjuvant immunotherapy were analyzed, including 60 (26.1%) with COPD. Multivariate logistic regression demonstrated that COPD was a predictor for MPR after neoadjuvant immunotherapy [odds ratio (OR), 2.490; 95% confidence interval (CI), 1.295–4.912; *P* = 0.007]. NSCLC with COPD showed a down-regulation of HERV–H LTR-associating protein 2 (HHLA2), which was an immune checkpoint molecule, and the HHLA2^low^ group demonstrated the enrichment of CD8^+^CD103^+^ tissue-resident memory T cells (TRM) compared to the HHLA2^high^ group (11.9% vs. 4.2%, *P* = 0.013). Single-cell analysis revealed TRM enrichment in the MPR group. Similarly, NSCLC with COPD exhibited a higher proportion of CD8^+^CD103^+^TRM compared to NSCLC without COPD (11.9% vs. 4.6%, *P* = 0.040).

**Conclusions:**

The study identified NSCLC with COPD as a favorable lung cancer type for neoadjuvant immunotherapy, offering a new perspective on the multimodality treatment of this patient population. Down-regulated HHLA2 in NSCLC with COPD might improve the MPR rate to neoadjuvant immunotherapy owing to the enrichment of CD8^+^CD103^+^TRM.

**Trial registration:**

Approval for the collection and utilization of clinical samples was granted by the Ethics Committee of Shanghai Pulmonary Hospital (Approval number: K23-228).

**Supplementary Information:**

The online version contains supplementary material available at 10.1186/s12885-024-12137-5.

## Introduction

Lung cancer is a highly prevalent and deadly disease. Previous research has shown that the incidence rate of non-small cell lung cancer (NSCLC) patients with chronic obstructive pulmonary disease (COPD) could reach as high as 50.5%[1]. NSCLC with COPD was reported to have a worse survival prognosis compared to NSCLC without COPD [2]. Immune checkpoint inhibitors (ICI) have become a viable strategy in cancer therapy; however, The rate of response was merely 12.5% in unselected patients [3]. Programmed cell death ligand 1 (PD-L1), tumor mutational burden (TMB), and mismatch repair (MMR)–deficient/microsatellite instability were reported to predict immunotherapy efficacy [4]. Zhou et al. found that NSCLC with COPD patients with advanced stage receiving anti-programmed cell death 1 (PD1)/PD-L1 immunotherapy obtained a more prolonged progression-free survival (PFS) in contrast to NSCLC without COPD patients [5]. In addition, emphysema, a typical manifestation of COPD, was also associated with an improved response to immunotherapy in NSCLC patients [6–8]. Nevertheless, additional research is required to determine whether NSCLC with COPD patients continue to represent a predominant population that benefits from neoadjuvant immunotherapy.

HERV–H LTR-associating protein 2 (HHLA2), alternatively identified as B7-H7, was a recently unveiled addition to the B7 family considered an immune checkpoint [9]. HHLA2 exhibited broad expression in human malignancies, including lung and breast cancer. Given its immunosuppressive function, HHLA2 was identified as a promising target for human cancer immunotherapy [10]. Previous research demonstrated that HHLA2 exhibited primary expression on both tumor cell and antigen-presenting cell (APC) membranes [9–12], inhibiting human CD4^+^ and CD8^+^T cell proliferation and activation when T cell receptor signaling was present [9, 13, 14]. However, the specific role of HHLA2 in NSCLC with COPD treated by immunotherapy has not yet been elucidated.

Tissue-resident memory T cells (TRM) constituted a specific CD8^+^ memory T cell subset, known to generate a potent anti-tumor immune response, and were linked to improved patient outcomes [15]. TRM were characterized by their presence within the tissue and exhibited enrichment of immune checkpoints and cytotoxic molecules, suggesting their significant contribution to tumor immunity [16]. Furthermore, the accumulation of CD8^+^CD103^+^TRM within tumors was a promising indicator for forecasting the effectiveness of ICI [17]. Nevertheless, the specific role of CD8^+^CD103^+^TRM in the response of NSCLC with COPD to immunotherapy remains unclear.

The objective of this study was to investigate the effectiveness of neoadjuvant immunotherapy among NSCLC with COPD versus NSCLC without COPD, and further explored the potential mechanistic links.

## Patients and methods

### Clinical data collection

Four hundred twenty lung cancer patients who underwent neoadjuvant immunotherapy in Shanghai Pulmonary Hospital from November 2020 to January 2023 were included. The inclusion criteria comprised the followings: (I) diagnosed with NSCLC; (II) with precise postoperative pathologic response degree. The exclusion criteria consisted of the followings: (I) incomplete neoadjuvant immunotherapy information; (II) a history of other malignancies; (III) without pre-neoadjuvant immunotherapy pulmonary function. Ultimately, 230 NSCLC patients were encompassed. Clinical and pathologic data were collected for all patients. Major pathologic response (MPR) was characterized by the presence of residual viable tumor not exceeding 10% at the time of resection. The diagnosis of COPD was based on forced expiratory volume in one second (FEV1)/forced vital capacity (FVC) < 0.70.

### Clinical samples collection

The clinical samples used for HE staining, immunohistochemistry, and flow cytometry were obtained from Shanghai Pulmonary Hospital with the patient’s informed consent. Approval for the collection and utilization of clinical samples was granted by the Ethics Committee of Shanghai Pulmonary Hospital (Ethical approval number: K23-228).

### RNA sequencing data

Forty NSCLC fresh-frozen specimens from our cohort were subjected to RNA sequencing analysis. The samples were processed to extract total RNA to assess its quality. RNA was further quantified and amplified. Subsequently, amplified RNA was fragmented and processed. Raw RNA sequencing data were further scanned. Additionally, RNA sequencing data of 1041 NSCLC tissues were obtained from The Cancer Genome Atlas (TCGA) database, including 175 samples with post-bronchodilator pulmonary function. Differential gene analysis was conducted using the Deseq2 package in the R software (https://www.r-project.org/) for both our cohort and the 175 TCGA samples data. Genes with an absolute value of Log_2_FC > 2 and P value < 0.05 were regarded as differentially expressed. The volcanic map was generated using the online platform (https://www.bioinformatics.com.cn), last accessed on 20 Feb 2023, to visualize the differential gene. The Venn diagram was plotted using Venny2.1.0 (https://bioinfogp.cnb.csic.es/tools/venny/index.html) to identify overlapping differential genes.

Immunologic gene sets from the MSigDB database (https://www.gsea-msigdb.org/gsea/msigdb/) and the immunologic infiltration analysis were analyzed using TCGA RNA sequencing data [18].

### Single-cell sequencing analysis

Single-cell data of patients receiving neoadjuvant immunotherapy from the Gene Expression Omnibus (GEO) dataset (GSE173351) were collected and analyzed [19]. After quality control, 42,678 T cells were remained for further analysis, and 15 distinct cell clusters were identified.

### HE staining

Paraffin-embedded slices of NSCLC samples were prepared and processed through dewaxing, dehydration, hematoxylin, and eosin staining. After dehydration, the slices were sealed with neutral gum. The extent of tumor cell necrosis was evaluated and recorded using an optical microscope. Based on the ratio of the tumor necrotic area to the entire area, we have divided it into four score grades as follows: score 1: 0-25%; score 2: 26-50%; score 3: 51-75%; score 4: 76-100%.

### Immunohistochemistry

Paraffin-embedded NSCLC samples underwent dewaxing and hydration. 3% H_2_O_2_ was used to block endogenous peroxidase activity. Antigen retrieval was conducted through microwave heating in a citrate buffer. The slices were incubated overnight with an anti-HHLA2 antibody. Subsequently, a secondary antibody labeled with horseradish peroxidase (HRP) was applied. The visualization of HHLA2 expression was achieved through DAB color rendering, and nuclear restaining was performed. The staining intensity for HHLA2 was evaluated to determine its expression in cells, and quantitative protein analysis was carried out using Image J software.

### Immunofluorescence

Paraffin slices were dewaxed and dehydrated, followed by antigen retrieval using citrate buffers. The slices were sealed with serum and double stained with primary antibodies against CD8 and CD103. Species-specific secondary antibodies labeled with HRP were applied, followed by DAPI nuclear restaining. Microscopic observation and quantitative cell analysis using Image J software were performed.

### Flow cytometry

Tumor samples were immediately minced and digested using collagenase IV in a 37℃ water bath. To achieve a single-cell suspension, the cell suspension obtained was filtered using a 70 μm filter. The single-cell suspension was processed through gradient centrifugation using a Percoll lymphocyte isolation solution to separate immune cells. The isolated immune cells were further lysed to remove red blood cells and stored at -80℃ for later use. For flow cytometry analysis, the cells were subsequently stained with fluorescent-labeled antibodies against live/dead (APCCY7), CD3 (BV510), CD4 (BB700), CD8 (BV605), CD103 (FITC), PD1 (APC), granzyme B (GZMB) (BV421), and interferon gamma (IFNG) (PE). The stained cells were then examined through the CytoFLEX system, and the acquired data were analyzed with FlowJo V10.6.2 software.

### Statistical analysis

Statistical analyses were performed utilizing R software and GraphPad Prism9. Logistic regression analysis was used to investigate the determinants of neoadjuvant immunotherapy efficacy. The rates between the two groups were compared using either the Chi-square test or Fisher’s exact test. Student’s t-test was used to analyze normally distributed continuous variables, while non-normally distributed continuous variables were assessed employing a nonparametric test. The statistical significance threshold was set at *P* < 0.05, and all analyses were performed using a two-tailed approach.

## Results

### NSCLC with COPD patients obtained a higher MPR rate to neoadjuvant immunotherapy

This study encompassed 230 NSCLC patients who underwent neoadjuvant immunotherapy. The median age was 64 years old [interquartile range (IQR), 58.0–68.0], with the majority of patients being male (*n* = 214, 93.0%) and smokers (*n* = 179, 77.8%). There were 60 (26.1%) NSCLC with COPD patients. NSCLC with COPD exhibited similar gender (*P* = 0.077), age (*P* = 0.072), and PD-L1 expression levels (*P* = 0.748) as NSCLC without COPD patients (Table 1).

**Table 1:** Basic information of NSCLC without COPD and NSCLC with COPD patients

Characteristics	Total	NSCLC without COPD	NSCLC with COPD	P
	*n* = 230	*n* = 170	*n* = 60	
**Gender**				0.077
**Male**	214 (93.0)	155 (91.2)	59 (98.3)	
**Female**	16 (7.0)	15 (8.8)	1 (1.7)	
**Age**	64.0 [58.0;68.0]	64.0 [58.0;68.0]	65.0 [60.0;69.0]	0.072
**Pathology**				0.058
**LUAD**	59 (25.7)	49 (28.8)	10 (16.7)	
**LUSC**	135 (58.7)	92 (54.1)	43 (71.7)	
**Others**	36 (15.6)	29 (17.1)	7 (11.6)	
**Neoadjuvant regimen**				0.346
**Immunotherapy only**	1 (0.5)	1 (0.6)	0 (0.0)	
**Immuno + Chemotherapy**	214 (93.0)	157 (92.4)	57 (95.0)	
**Immuno + Targeted/Anti-vascular therapy**	8 (3.5)	5 (2.9)	3 (5.0)	
**Immuno + Chemo + Targeted therapy**	7 (3.0)	7 (4.1)	0 (0.0)	
**Pathologic response**				0.004
**Non-MPR**	110 (47.8)	91 (53.5)	19 (31.7)	
**MPR**	120 (52.2)	79 (46.5)	41 (68.3)	
**Clinical stage**				0.044
**I/II**	65 (28.3)	42 (24.7)	23 (38.3)	
**III**	165 (71.7)	128 (75.3)	37 (61.7)	
**Treatment cycles**	3.0 [2.0;4.0]	3.0 [2.0;4.0]	3.0 [2.0;4.0]	0.778
**Smoking history**				0.227
**No**	15 (6.5)	14 (8.2)	1 (1.7)	
**Yes**	179 (77.8)	129 (75.9)	50 (83.3)	
**Unknown**	36 (15.7)	27 (15.9)	9 (15.0)	
**FEV1**	2.4 [1.9;2.8]	2.5 [2.2;2.9]	1.9 [1.6;2.2]	<0.001
**FEV1%**	87.7 [74.3;98.1]	92.1 [83.3;102.0]	67.3 [56.2;75.6]	<0.001
**PD-L1 expression**				0.748
**PD-L1 < 50%**	123 (53.5)	93 (54.7)	30 (50.0)	
**PD-L1 ≥ 50%**	29 (12.6)	20 (11.8)	9 (15.0)	
**Unknown**	78 (33.9)	57 (33.5)	21 (35.0)	

Values are presented as median [IQR1-IQR3] or n (%); IQR, interquartile range; NSCLC, non-small cell lung cancer; MPR, major pathologic response; LUSC, lung squamous cell carcinoma; LUAD, lung adenocarcinoma; COPD, chronic obstructive pulmonary disease; FEV1, forced expiratory volume in one second; PD-L1, programmed cell death ligand 1

**Fig. 1:**
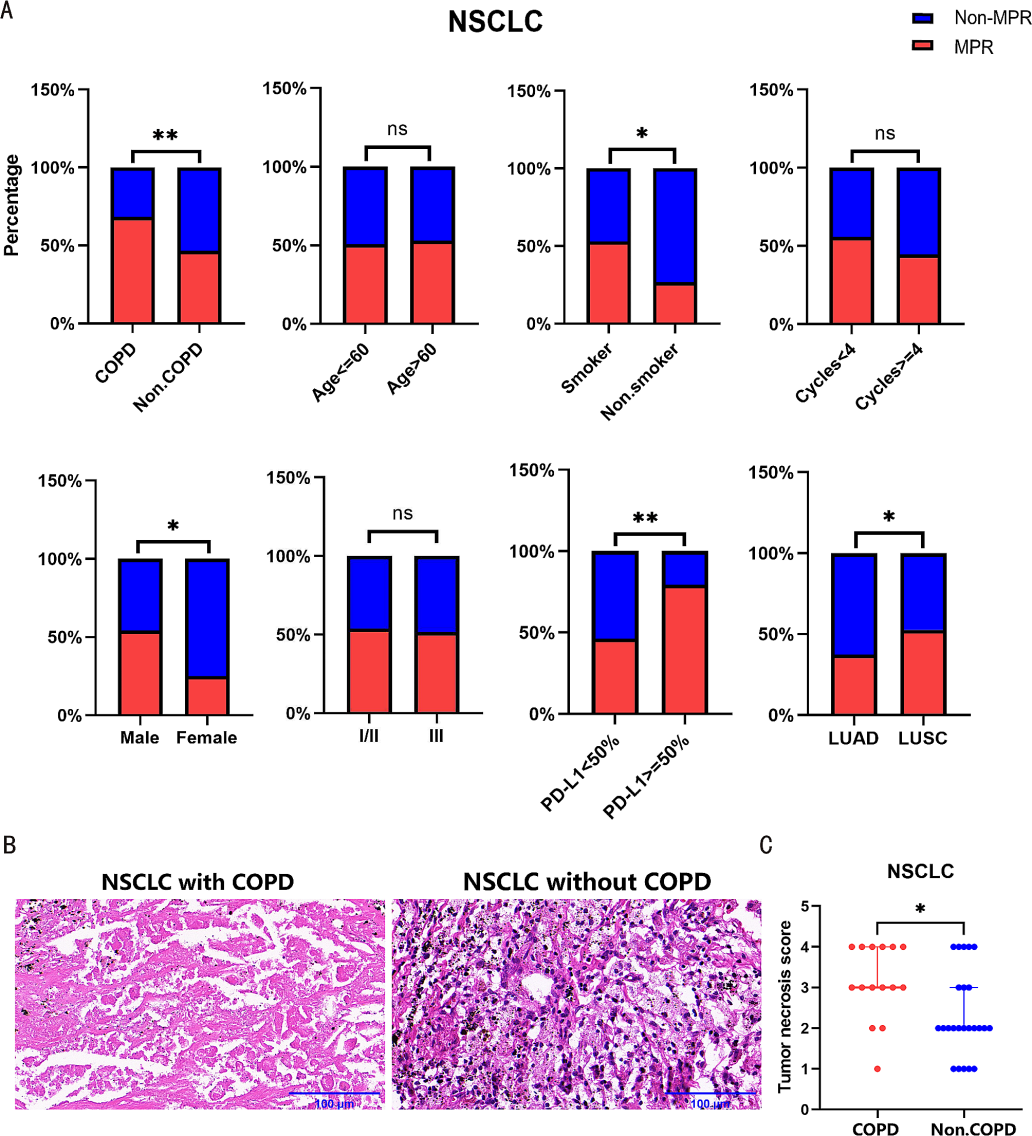
NSCLC with COPD patients obtained a higher MPR rate to neoadjuvant immunotherapy. (**A**) The efficacy to neoadjuvant immunotherapy in NSCLC patients with different COPD status, age, smoking history, gender, clinical stage, PD-L1 expression, treatment cycles, and pathology were compared. (**B**) Representative pictures of HE staining of patients received neoadjuvant immunotherapy. Left: NSCLC with COPD; Right: NSCLC without COPD. (**C**) Statistical chart of HE staining in NSCLC with COPD versus NSCLC without COPD patients received neoadjuvant immunotherapy. Scale bars, 100 μm.*P < 0.05, **P < 0.01, ***P < 0.001, ****P < 0.0001

In addition, pathologic results showed that 120 (52.2%) patients achieved MPR following neoadjuvant immunotherapy. There were no noteworthy disparities in the MPR rates between different age groups and clinical stages in NSCLC patients. NSCLC with COPD had a higher MPR rate to neoadjuvant immunotherapy compared to NSCLC without COPD (68.3% vs. 46.5%, *P* = 0.004). Additionally, patients with a smoking history, higher PD-L1 expression or lung squamous cell carcinoma (LUSC) responded better to immunotherapy (Table 2; Fig. 1A).

**Table 2:** Factors affecting neoadjuvant immunotherapy efficacy of NSCLC patients

Characteristics	*n* = 230	MPR 120 (52.2)	Univariate			Multivariate		
			OR	95%CI	P	OR	95%CI	P
**Gender**								
** Male**	214	116 (54.2)	reference			reference		
** Female**	16	4 (25.0)	0.282	0.077–0.837	0.033	0.273	0.062–0.981	0.059
**Age**								
** Age ≤ 60**	77	39 (50.7)	reference					
** Age > 60**	153	81 (53.0)	1.096	0.633–1.898	0.743			
**Pathology**								
** LUSC**	135	71 (52.6)	reference			reference		
** LUAD**	59	22 (37.3)	0.536	0.283–0.996	0.051	0.690	0.340–1.384	0.298
** Others**	36	27 (75.0)	2.704	1.222–6.487	0.018	3.478	1.484–8.842	0.006
**Clinical stage**								
** III**	165	85 (51.5)	reference					
** I/II**	65	35 (53.8)	1.098	0.618–1.959	0.750			
**Treatment cycles**								
** Cycles < 4**	156	87 (55.8)	reference					
** Cycles ≥ 4**	74	33 (44.6)	0.664	0.378–1.158	0.150			
**Smoking history**								
** Yes**	179	95 (53.1)	reference					
** No**	15	4 (26.7)	0.322	0.087–0.980	0.060			
** Unknown**	36	21 (58.3)	1.238	0.603–2.594	0.564			
**COPD**								
** No**	170	79 (46.5)	reference			reference		
** Yes**	60	41 (68.3)	2.486	1.350–4.708	0.004	2.490	1.295–4.912	0.007
**PD-L1 expression**								
** PD-L1 < 50%**	123	57 (46.3)	reference			reference		
** PD-L1 ≥ 50%**	29	23 (79.3)	4.439	1.785–12.703	0.002	5.235	1.976–16.045	0.002
** Unknown**	78	40 (51.3)	1.219	0.691–2.156	0.495	1.229	0.661–2.293	0.515

Values are presented as n (%); NSCLC, non-small cell lung cancer; MPR, major pathologic response; OR, odds ratio; CI, confidence interval; LUSC, lung squamous cell carcinoma; LUAD, lung adenocarcinoma; COPD, chronic obstructive pulmonary disease; PD-L1, programmed cell death ligand 1

Univariate analysis showed that COPD exhibited a significant association with MPR [odds ratio (OR), 2.486; 95% confidence interval (CI), 1.350–4.708; *P* = 0.004], along with pathology, PD-L1 expression and gender. Multivariate logistic regression analysis confirmed that COPD remained as an predictor associated with a better response to neoadjuvant immunotherapy (OR, 2.490; 95%CI, 1.295–4.912; *P* = 0.007) (Table 2). Furthermore, HE staining of pathologic specimens revealed more pronounced tumor cell necrosis in NSCLC with COPD (Fig. 1B-C).

### NSCLC with COPD showed a down-regulation of HHLA2

**Fig. 2:**
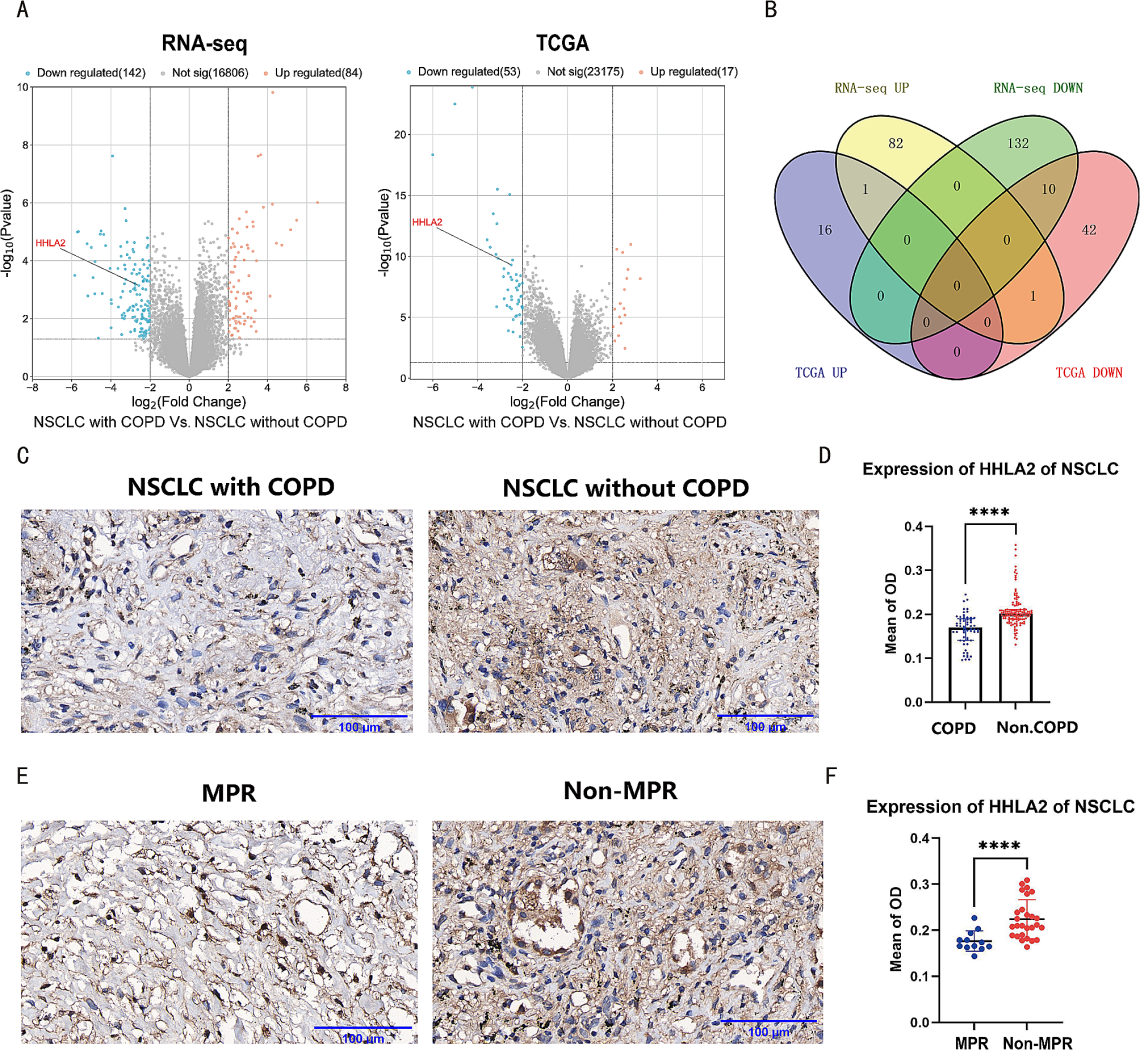
NSCLC with COPD showed a down-regulation of HHLA2. (**A**) Left: Volcanic map of differential genes in 40 samples paired with NSCLC with COPD and NSCLC without COPD. Right: Volcanic map of differential genes in 175 TCGA samples paired with NSCLC with COPD and NSCLC without COPD. The blue and orange dots represent down-regulated and up-regulated genes, respectively. (**B**) Venn diagram of the intersection of differential genes mentioned above. (**C**) Representative image of HHLA2 immunohistochemical staining. Left: NSCLC with COPD; Right: NSCLC without COPD. (**D**) Statistical chart of HHLA2 immunohistochemical staining in NSCLC with COPD versus NSCLC without COPD. (**E**) Representative images of HHLA2 immunohistochemical staining. Left: the MPR group; Right: the non-MPR group. (**F**) Statistical chart of HHLA2 immunohistochemical staining in the MPR group versus the non-MPR group. Scale bars, 100 μm. *P < 0.05, **P < 0.01, ***P < 0.001, ****P < 0.0001

Differential gene analysis was conducted using RNA sequencing data of 40 samples from our cohort, including NSCLC with COPD (*n* = 12) and NSCLC without COPD (*n* = 28). 226 differential genes were detected, comprising 142 down-regulated and 84 up-regulated genes (Fig. 2A). Differential gene analysis was also performed using RNA sequencing data from 175 TCGA samples with post-bronchodilator pulmonary function, including NSCLC with COPD (*n* = 65) and NSCLC without COPD (*n* = 110). Seventy differential genes were found, including 53 down-regulated and 17 up-regulated genes (Fig. 2A). By comparing the two datasets, we discovered ten down-regulated (HHLA2, BRINP1, CA10, CEACAM8, CLDN6, HNF4A, LGALS4, OLFM4, SERPINA4 and UGT2B15) and one up-regulated (S100A7) genes that were consistently differentially expressed (Fig. 2B). And then, the down-regulated HHLA2 was selected for further investigation due to its relevance to tumor immunity. Immunohistochemical staining of tissue slices confirmed that HHLA2 expression was lower in NSCLC with COPD (*n* = 20) compared to NSCLC without COPD (*n* = 42) (Fig. 2C-D). In addition, immunohistochemical staining showed that HHLA2 expression was also lower in the MPR group (Fig. 2E-F).

### Down-regulated HHLA2 was associated with CD8^+^CD103^+^TRM enrichment

**Fig. 3:**
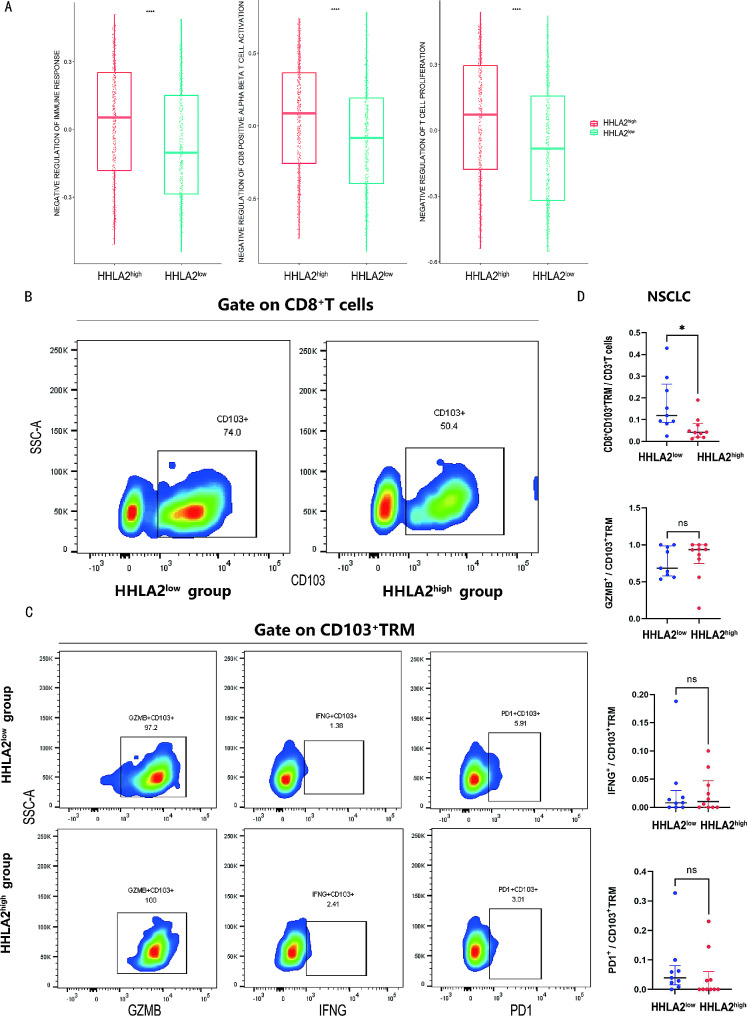
Down-regulated HHLA2 was associated with CD8^+^CD103^+^TRM enrichment. (**A**) Comparison of negative regulation of immune response, T cell proliferation, and CD8^+^T cell activation gene sets from the MSigDB database based on TCGA in the HHLA2^low^ and HHLA2^high^ group. (**B**) Representative flow cytometry chart of CD8^+^CD103^+^TRM in the HHLA2^low^ versus HHLA2^high^ group. (**C**) Representative flow cytometry chart of GZMB, IFNG, PD1 among CD8^+^CD103^+^TRM in the HHLA2^low^ versus HHLA2^high^ group. (**D**) Statistical maps of CD8^+^CD103^+^TRM proportion and function in the HHLA2^low^ versus HHLA2^high^ group. *P < 0.05, **P < 0.01, ***P < 0.001, ****P < 0.0001

TCGA RNA sequencing data of 1041 samples were analyzed and categorized into HHLA2^high^ (*n* = 520) and HHLA2^low^ (*n* = 521) groups according to the median expression level of HHLA2. Using the MSigDB database to perform immune function scoring on the two groups, the HHLA2^high^ group exhibited higher scores in gene sets related to inhibiting immune response, T cell proliferation, and CD8^+^T cell activation than the HHLA2^low^ group (Fig. 3A). These results suggested that HHLA2 may be linked to the tumor microenvironment.

To explore the correlation between HHLA2 and the tumor microenvironment, 19 samples assessed for HHLA2 expression via immunohistochemistry were further divided into two groups: HHLA2^high^ (*n* = 10) and HHLA2^low^ (*n* = 9) based on median HHLA2 expression for the flow cytometry analysis. The flow cytometry analysis showed that a greater proportion of CD8^+^CD103^+^TRM within CD3^+^ cells was observed in the HHLA2^low^ group compared to the HHLA2^high^ group (11.9% vs. 4.2%, *P* = 0.013). However, there were no statistically significant differences in the expression levels of GZMB, IFNG, and PD1 in CD8^+^CD103^+^TRM between the two groups (Fig. 3B-D). These findings demonstrated that down-regulated HHLA2 was associated with CD8^+^CD103^+^TRM enrichment in the tumor microenvironment.

### Enrichment of CD8^+^CD103^+^TRM in the MPR group

**Fig. 4:**
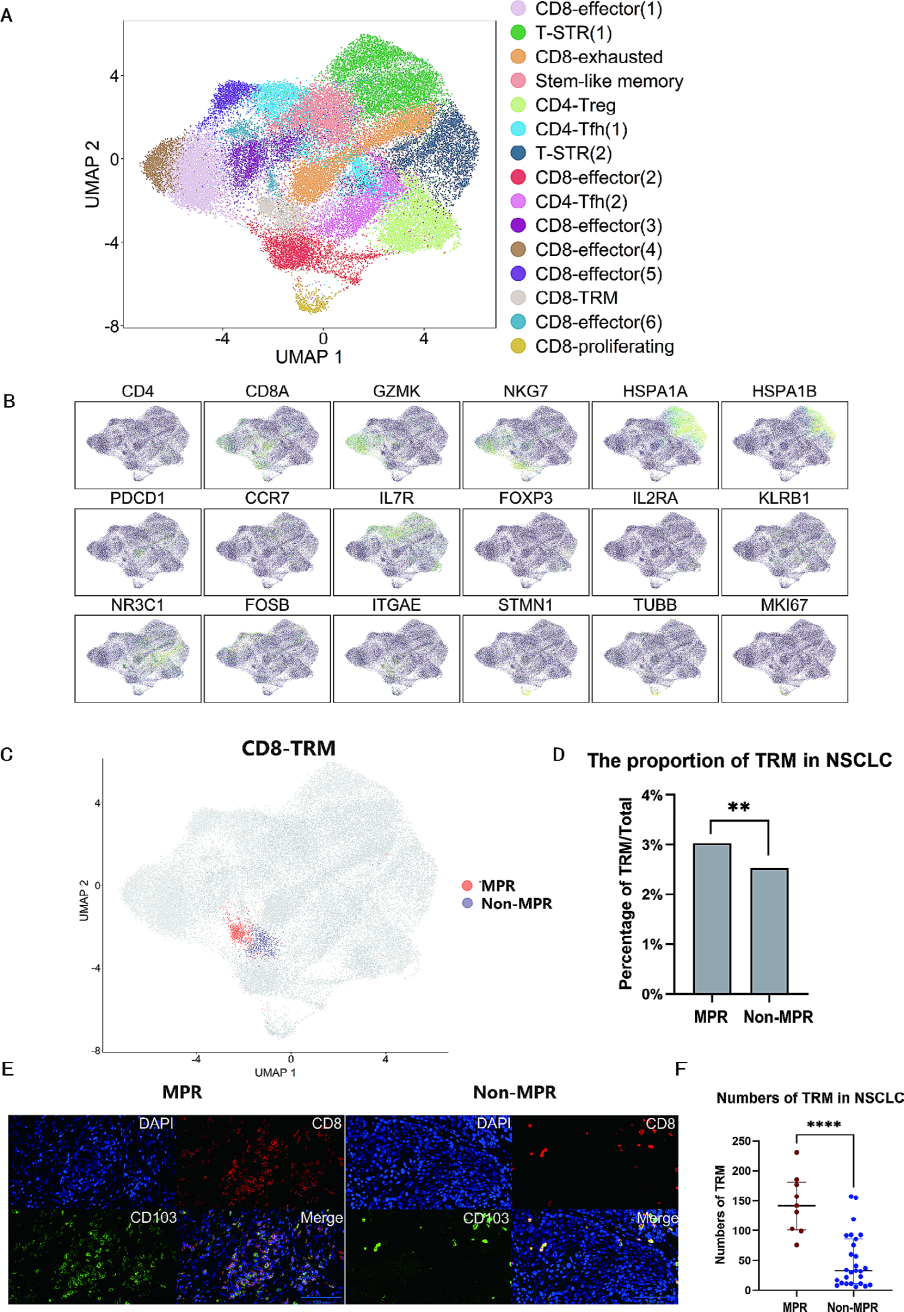
The enrichment of CD8^+^CD103^+^TRM in the MPR group. (**A**) Single-cell analysis divided 42,678 cells into 15 sub-clusters. Treg, regulatory T cells; T-STR, stress response state T cells; Tfh, T follicular helper cells. (**B**) Expression of T cell subset-defining genes. (**C**) Distribution image of CD8^+^CD103^+^TRM in the MPR versus non-MPR group. (**D**) Statistical chart of the proportion of CD8^+^CD103^+^TRM in single-cell analysis in the MPR versus non-MPR group. (**E**) Representative images of TRM in immunofluorescence analysis. Left: the MPR group; Right: the non-MPR group. (**F**) Statistical chart of TRM of immunofluorescence analysis in the MPR versus non-MPR group. Scale bars, 100 μm. *P < 0.05, **P < 0.01, ***P < 0.001, ****P < 0.0001

Single-cell data from six NSCLC patients, including the MPR (*n* = 3) and non-MPR group (*n* = 3), were analyzed. The study identified 42,678 T cells and 15 distinct cell clusters, including CD8^+^ effector T cells (GZMK, NKG7), stress response state T cells (T-STR) defined by the elevated expression of heat shock genes (HSPA1A, HSPA1B) [20], CD8^+^ proliferating T cells (TUBB, STMN1), stem-like memory T cells (CCR7, IL7R), CD4^+^ T follicular helper cells (Tfh) (KLRB1, NR3C1), CD4^+^ regulatory T cells (Treg) (FOXP3, IL2RA), CD8^+^TRM (CD8A, ITGAE known as CD103), and CD8^+^ exhausted T cells (CD8A, PDCD1) (Fig. 4A-B). Among all the T cells, 1181 cells were identified as CD8^+^CD103^+^TRM. The MPR group comprised 20,271 single cells, including 614 CD8^+^CD103^+^TRM, while the non-MPR group comprised 22,407 single cells, with 567 CD8^+^CD103^+^TRM (Fig. 4C). Notably, a higher proportion of CD8^+^CD103^+^TRM was observed in the MPR group in contrast to the non-MPR group (3.0% vs. 2.5%, *P* = 0.002) (Fig. 4D). Differences of additional typical cell clusters between the MPR and non-MPR groups were shown in supplementary Figure S1. Immunofluorescence analysis further demonstrated the enrichment of TRM in the MPR group as opposed to the non-MPR group (Fig. 4E-F). These results implied that the enrichment of TRM was correlated with MPR.

### NSCLC with COPD was featured by CD8^+^CD103^+^TRM enrichment

**Fig. 5:**
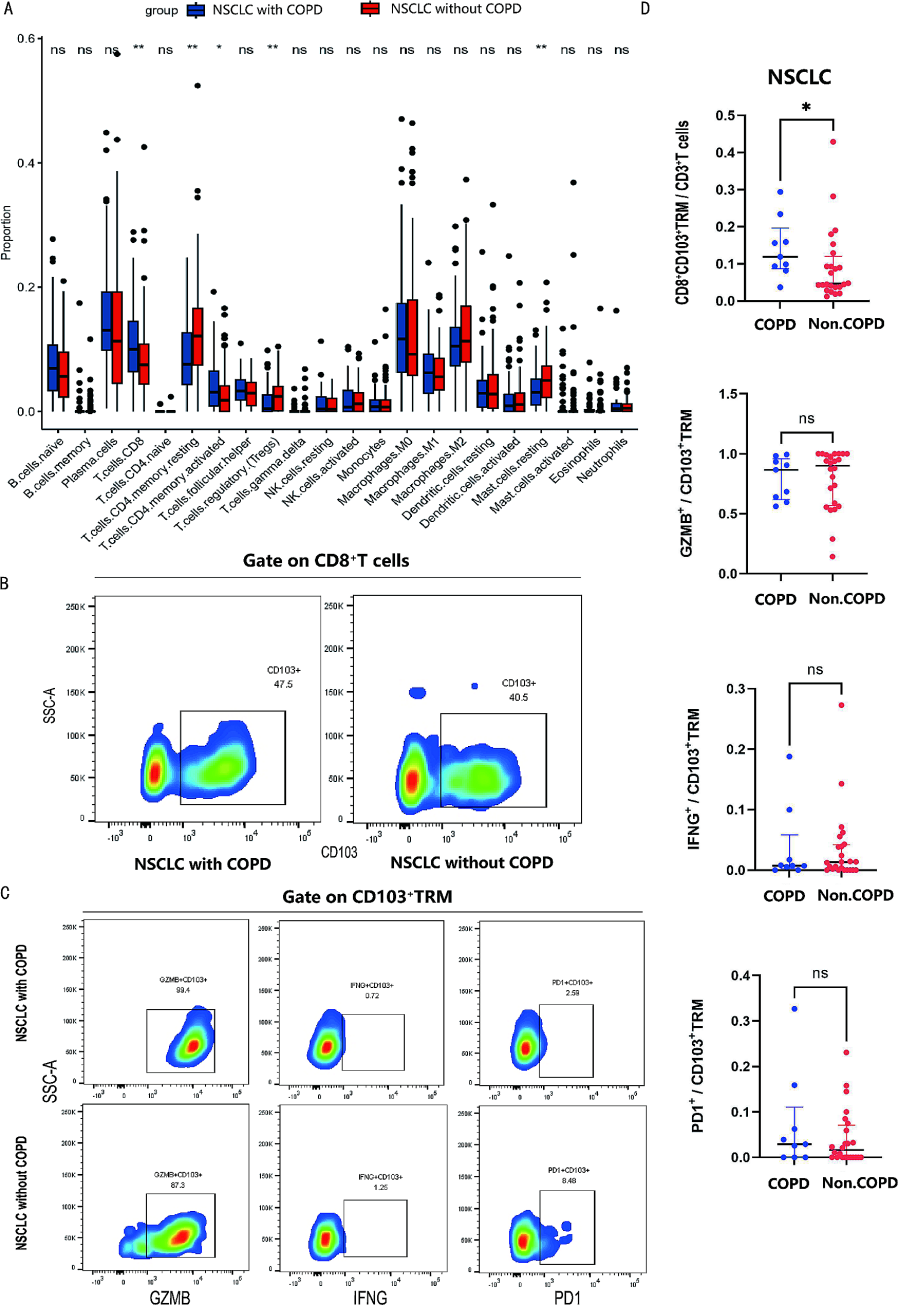
NSCLC with COPD was featured by CD8^+^CD103^+^TRM enrichment. (**A**) Immune infiltration analysis in NSCLC with COPD versus NSCLC without COPD via CIBERSORT based on TCGA data. (**B**) Representative flow cytometry chart of CD8^+^CD103^+^TRM in NSCLC with COPD versus NSCLC without COPD. (**C**) Representative flow cytometry chart of GZMB, IFNG, PD1 among CD8^+^CD103^+^TRM in NSCLC with COPD versus NSCLC without COPD. (**D**) Statistical maps of CD8^+^CD103^+^TRM proportion and function in NSCLC with COPD versus NSCLC without COPD. *P < 0.05, **P < 0.01, ***P < 0.001, ****P < 0.0001

RNA sequencing data of 175 TCGA samples with post-bronchodilator pulmonary function were categorized into two groups: NSCLC with COPD (*n* = 65) and NSCLC without COPD (*n* = 110). Immunologic infiltration analysis through CYBERSORT algorithm based on the data showed that NSCLC with COPD exhibited an increased proportion of CD8^+^T cells and a decreased proportion of Treg compared to NSCLC without COPD (Fig. 5A). Flow cytometry analysis was used further to compare the proportion and function of T cells and revealed a greater proportion of CD8^+^CD103^+^TRM within CD3^+^T cells in NSCLC with COPD (*n* = 9) compared to NSCLC without COPD (*n* = 24) (11.9% vs. 4.6%, *P* = 0.040). Nevertheless, there were no statistically significant differences among the expression of GZMB, IFNG, and PD1 among CD8^+^CD103^+^TRM cells between two groups (Fig. 5B-D). These outcomes validated that CD8^+^CD103^+^TRM were accumulated in NSCLC with COPD.

## Discussion

This study was the first to identify NSCLC with COPD patients as an advantaged population for neoadjuvant immunotherapy. Down-regulated HHLA2 in NSCLC with COPD might improve the response to neoadjuvant immunotherapy by means of the enrichment of CD8^+^CD103^+^TRM.

Previous research have demonstrated that lung cancer patients with COPD had certain characteristics that suggested they may benefit from immunotherapy, such as high TMB, high tumor neo-antigen burden (TNB), high mutation frequencies of immune-related genes like LRP1B and PREX2, and low mutation frequency of EGFR [21, 22]. Furthermore, it has been reported that advanced NSCLC with COPD had a better overall survival (OS) and PFS compared to NSCLC without COPD when treated by immunotherapy [23]. Building upon these findings, our study further investigated the therapeutic effectiveness of neoadjuvant immunotherapy, demonstrating that NSCLC with COPD could obtain a better MPR rate than NSCLC without COPD.

The immune checkpoint expression profiles in the airway tissue of COPD showed the down-regulation of HHLA2 and lymphocyte activation gene-3 (LAG3), as well as the up-regulation of PD1 compared to health control [24]. Our study, through sequencing and immunohistochemical analysis, showed that NSCLC with COPD exhibited down-regulation of HHLA2 expression in the tumor immune microenvironment, particularly in tumor cells. HHLA2 was reported to disrupt anti-tumor immunity and inhibit immune surveillance [25]. In addition, Zhou et al. demonstrated that the co-expression of HHLA2 and PD-L1 negatively impacted the prognosis of clear cell renal cell carcinoma patients, suggesting these patients may derive advantages from the combined inhibition of both PD-L1 and HHLA2 [26]. Similar findings regarding the dual blockade of PD-L1 and HHLA2 have also been reported in spinal chordoma patients [27]. Our study further confirmed the down-regulation of HHLA2 in NSCLC patients achieving MPR.

Previous reports showed that HHLA2 initially inhibited T-cell proliferation and cytokine production by inhibiting phosphatidylinositol-3 kinases (PI3K)-protein kinase B (PKB/AKT) signaling [11, 28]. When HHLA2 was down-regulated, this inhibitory effect was weakened, thus promoting the anti-tumor immune system. Additionally, HHLA2/Killer Cell Immunoglobulin Like Receptor, Three Ig Domains And Long Cytoplasmic Tail 3 (KIR3DL3) signaling was reported to participate in modulating tissue-resident and innate-like T cells [29]. Our study confirmed that down-regulated HHLA2 was associated with CD8^+^CD103^+^TRM enrichment.

TRM were regarded as a foundation for effective neoadjuvant immunotherapy. These cells were believed to rapidly initiate the immune response against the tumor following treatment [30, 31]. In addition, previous research confirmed TRM infiltrating NSCLC tumors as activated cytotoxic cells, significantly contributing to anti-tumor immunity [17]. Similarly, when co-cultured with breast cancer cells, it was found that CD8^+^CD69^+^CD103^+^TRM exhibited higher levels of IFNG and tumor necrosis factor-alpha (TNF-α) compared to CD8^+^CD69^+^CD103^−^T cells, further mediating a more significant tumor-killing effect [32]. The augmentation of tumor-resident memory T cells correlated with improved survival outcomes in melanoma patients undergoing immunotherapy [33, 34]. In addition, TRM could convert “cold” into “hot” tumors, thereby improving the effectiveness of immunotherapy in gastrointestinal tumors [35]. Our study demonstrated the enrichment of TRM in the MPR group. This could be explained by the restoration of cytotoxic function in TRM following ICI therapy, thereby enhancing the efficacy of immunotherapy.

The potential advantages of neoadjuvant immunotherapy for NSCLC with COPD may be attributed to the long-term chronic inflammation and remodeling of the pulmonary immuno-microenvironment. The increased proportion of CD8^+^T cells and T cell functional exhaustion were identified as critical immunophenotypic features in COPD [36–38]. Our study further validated the distribution of TRM in NSCLC with COPD versus NSCLC without COPD, and confirmed the enrichment of TRM in NSCLC with COPD.

Our study existed several limitations. First, the retrospective analysis of neoadjuvant immunotherapy’s effectiveness was hindered by patient bias towards LUSC, smoking history, and preference for combined immunotherapy and chemotherapy, complicating the differentiation between their respective efficacies and analysis based on smoking status. Second, due to the difficulty in obtaining NSCLC samples from patients who had concurrent COPD and underwent neoadjuvant immunotherapy, our study mainly utilized NSCLC surgical samples for analyses. Third, this study was lack of genomic information and the detailed mechanisms underlying the study results required further validation.

## Conclusions

In summary, the study identified NSCLC with COPD patients as an advantaged population for neoadjuvant immunotherapy, offering a new perspective on the multimodality treatment of these patients. Down-regulated HHLA2 in NSCLC with COPD might improve the MPR rate to neoadjuvant immunotherapy by means of the enrichment of CD8^+^CD103^+^TRM.

### Electronic supplementary material

Below is the link to the electronic supplementary material.

Supplementary Material 1

## Data Availability

The datasets used and/or analysed during the current study are available from the corresponding author on reasonable request.

## Abbreviations

COPD: chronic obstructive pulmonary disease
NSCLC: non-small cell lung cancer
MPR: major pathologic response
OR: odds ratio
CI: confidence interval
HHLA2: HERV–H LTR-associating protein 2
TRM: tissue-resident memory T cells
ICI: immune checkpoint inhibitors
PD-L1: programmed cell death ligand 1
TMB: tumor mutational burden
MMR: mismatch repair
PD1: programmed cell death 1
PFS: progression-free survival
APC: antigen-presenting cells
FEV1: forced expiratory volume in one second
FVC: forced vital capacity
TCGA: The Cancer Genome Atlas
GEO: Gene Expression Omnibus
HRP: horseradish peroxidase
GZMB: granzyme B
IFNG: interferon gamma
IQR: interquartile Range
TNB: tumor neo-antigen burden
OS: overall survival
LAG3: lymphocyte activation gene-3
PI3K: phosphatidylinositol-3 kinases
PKB: protein kinase B
KIR3DL3: Killer Cell Immunoglobulin Like Receptor, Three Ig Domains And Long Cytoplasmic Tail 3
LUSC: lung squamous cell carcinoma
LUAD: lung adenocarcinoma
T-STR: stress response state T cells
Tfh: T follicular helper cells
Treg: regulatory T cells
TNF-α: tumor necrosis factor-alpha
