# Supplementary material for: Down-regulated HHLA2 enhances neoadjuvant immunotherapy efficacy in patients with non-small cell lung cancer (NSCLC) with chronic obstructive pulmonary disease (COPD)
Source: BMC Cancer. 2024 Mar 29;24:396. doi: 10.1186/s12885-024-12137-5 (PMC10979619; doi:10.1186/s12885-024-12137-5)
Supplement: Supplementary file 1 — Supplementary Material 1 [file 12885_2024_12137_MOESM1_ESM.docx]

**Supplementary Figure S1**


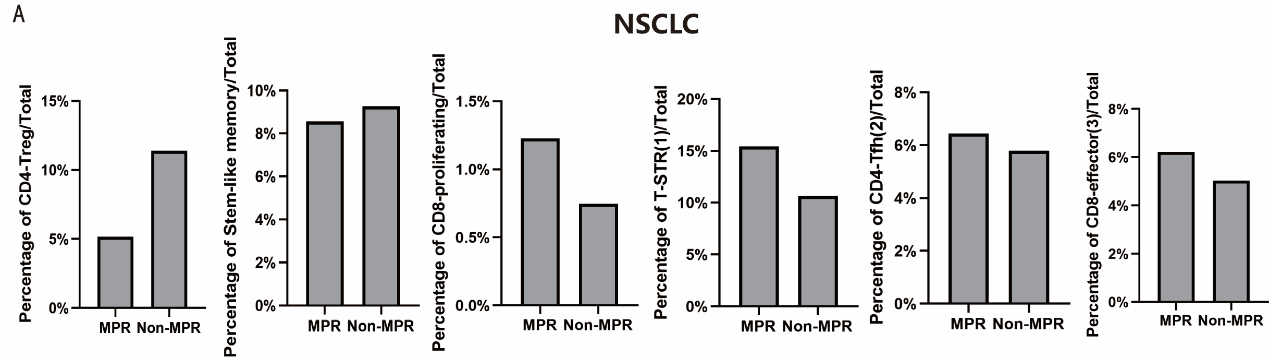


Figure S1: Differences of other cell clusters between the MPR and the non-MPR groups. Treg, regulatory T cells; T-STR, stress response state T cells; Tfh, T follicular helper cells.
